# Rare developmental abnormalities of thyroid gland, especially multiple ectopia: A review and our experience

**DOI:** 10.4103/0972-3919.78248

**Published:** 2010

**Authors:** Anuj Jain, Sujata Pathak

**Affiliations:** Department of Nuclear Medicine and PET/CT, Vijaya Diagnostic Center Pvt. Ltd., Hyderabad, India

**Keywords:** Developmental abnormalities of thyroid, dual ectopia, triple ectopia, thyroid scan

## Abstract

**Background:**

Developmental structural abnormalities of the thyroid gland are relatively rare. There are scanty reports of hemiagensis, dual and triple ectopia of the thyroid in the literature

**Materials and Methods:**

We did a retrospective analysis of 236 patients referred to us for Tc-99m Pertechnetate thyroid scan over period of four months (May 2010 to Sept 2010). Twenty of these 236 patients aged less than 20 years found to have developmental abnormality of the thyroid gland on thyroid scan. Diagnosis was correlated with anatomical imaging (USG/CT scan), fine needle aspiration cytology (FNAC) and histopathology.

**Results:**

Out of the 20 patients, 8 were diagnosed with thyroglossal cyst, 4 with ectopic thyroid gland, 4 with dual ectopia, two had agenesis of thyroid gland, one case each with hemiagenesis and triple ectopia.

**Conclusion:**

The study has emphasized the indispensable role of Tc-99m Pertechnetate thyroid scan in the evaluation of midline neck swellings of childhood and diagnosing developmental anomalies of thyroid gland.

## INTRODUCTION

The thyroid gland develops from the foregut and descends by a circuitous route to its normal cervical position. Its original position is marked by the foramen caecum at the junction of anterior two-thirds and posterior one-third of the tongue. The first evidence of its presence is at about 4 weeks of gestational age as an evagination between the first and second pharyngeal pouches. This evagination lengthens to form a tube, which descends inferiorly and anteriorly to pass anterior to the hyoid bone. It then loops round and behind the hyoid before continuing its descent in the neck to finally form two lateral buds; these will become the lateral lobes. They fuse with the ultimobranchial body which supplies the cells that will become the parafollicular cells. Thus follicular and parafollicular cells have different lineage. The pathway from the pharynx to the anterior neck is marked by the thyroglossal duct. The thyroglossal duct loses its tubular structure and by the sixth week atrophies. The follicular cells are able to trap iodine by the twelfth week and hormone production occurs soon after.[1] In the majority of cases the cause of thyroid ectopia remains unclear. Mutation in thyroid transcription factor-2, which is required for the downward migration of the thyroid gland, has been proposed as a possible mechanism.[2]

Developmental abnormalities (excluding biochemical abnormalities) of the thyroid gland can be divided into three major groups: (1) agenesis of thyroid gland, which is an important cause of neonatal hypothyroidism; (2) dysgenesis of the thyroid; and (3) abnormalities due to persistence of the thyroglossal duct.

Dysgenesis can be in the form of hemiagenesis of the thyroid or as an ectopic thyroid. More than 100 cases of thyroid hemiagenesis have been reported.[3] The left lobe is absent in 80% of these patients. Often, the thyroid lobe that is present is enlarged. Both hyperthyroidism and hypothyroidism have been reported. Females are affected three times as often as males. Both benign and malignant nodules have been reported in this condition.[4] According to the site at which it is found the ectopic thyroid may be lingual, perihyoid, intratracheal, intraesophageal, mediastinal, or cardiac. The lingual site is by far most common location for functioning ectopic thyroid tissue. Lingual thyroid tissue is associated with an absence of the normal cervical thyroid in 70% of cases. It occurs much more commonly in women than in men. The diagnosis is usually made by the incidental discovery of a mass on the back of the tongue in an asymptomatic patient. The mass may enlarge and cause dysphagia, dysphonia, dyspnea, or a sensation of choking.[5] Hypothyroidism is often present and may cause the mass to enlarge and become symptomatic, but hyperthyroidism is very unusual. In women, the symptomatic lingual thyroid gland develops during puberty or early adulthood in most cases.[6] Suprahyoid and infrahyoid ectopic thyroid tissue is seen in a midline position above or below the hyoid bone. Hypothyroidism is commonly present because of the absence of a normal thyroid gland in most instances. An enlarging mass commonly occurs during infancy, childhood, or later life. Often, this mass is mistaken for a thyroglossal duct cyst because it is usually located in the same anatomic position.[7] If it is removed all thyroid tissue may be ablated, which has definite physiologic as well as possible medicolegal implications. To prevent total thyroid ablation, a thyroid scan or ultrasound examination must be performed in all cases of thyroglossal duct cyst before its removal so as to be certain that a normal thyroid gland is present. Multiple ectopia (dual and triple) of the thyroid gland is very rare and few cases have been reported in the literature.[8–16]

Both cysts and fistulas can develop along the course of the thyroglossal duct.[17] These cysts are the most common anomaly of thyroid development seen in clinical practice.[18] Knight *et al*. found that 52% of 146 children with an anterior neck swelling had thyroglossal cyst.[19] Normally, the thyroglossal duct becomes obliterated early in embryonic life but occasionally it persists as a cyst. Such lesions occur equally in males and females. They are seen at birth in about 25% of cases. Most appear in early childhood, while the rest, i.e., about one-third, become apparent only after the age of 30 years.[20] There is not enough thyroid tissue in a thyroglossal cyst to be imaged on radionuclide scintigraphy, thus allowing differentiation of a cyst from an ectopic thyroid. Cysts usually appear in the midline or just off the midline, between the isthmus of the thyroid and the hyoid bone. They commonly become repeatedly infected and may rupture spontaneously. When this complication occurs, a sinus tract or fistula forms and persists. Removal of a thyroglossal cyst or fistula requires excision of the central part of the hyoid bone and dissection of the thyroglossal tract up to the base of the tongue (the Sistrunk procedure) if recurrence is to be minimized. This procedure is necessary because the thyroglossal duct is intimately associated with the central part of the hyoid bone.[21]

## MATERIALS AND METHODS

In all patients in our series a thyroid scan was performed after intravenous injection of 70–150 MBq of 99mTc pertechnetate. Initial flow and blood pool images and a static image 20 min after injection were taken. Imaging was done using a dual-headed gamma camera (Hawkeye, GE Healthcare). Oblique and lateral views were also taken in selected case.

Findings of the scan were correlated with anatomical imaging in almost all cases and, in a few cases, with fine needle aspiration cytology (FNAC) or histopathology.

## RESULTS

In this retrospective analysis of 236 patients referred to us for thyroid scan over a 5-month period (May 2010 to Sept 2010), 41 patients were below 20 years of age. Of these 41 patients, 20 were diagnosed with some developmental abnormality of the thyroid gland: 8 patients were diagnosed with thyroglossal cyst, 4 with ectopic thyroid gland, 4 with dual ectopia, 2 with agenesis of thyroid gland, 1 with hemiagenesis, and 1 with triple ectopia [Table 1]. Thyroiditis, diffuse toxic goiter, simple goiter, and cold nodules were the other thyroid disorders found in the <20 years age-group. Developmental disorders were more common in the younger age-group (<10 years), while other disorders were more common in older children.

**Table 1:** Age, sex, and thyroid abnormality of the 20 patients

Age	Sex	Thyroid abnormality
6 years	F	Dual ectopia
10 years	F	Dual ectopia
20 years	F	Triple ectopia
10 years	F	Sublingual
7 years	F	Thyroglossal cyst
16 years	F	Thyroglossal cyst
2 years	F	Suprahyoid ectopic
3 months	F	Agenesis
18 years	F	Subhyoid ectopic
17 years	F	Hemiagenesis
5 years	M	Agenesis
15 years	F	Thyroglossal cyst
4 years	M	Thyroglossal cyst
14 years	F	Dual ectopia
10 years	F	Thyroglossal cyst
14 years	M	Dual ectopia
4 years	F	Lingual ectopic with normal thyroid
6 years	M	Thyroglossal cyst
17 years	F	Thyroglossal cyst
4 years	F	Thyroglossal cyst

The most common developmental disorder of the thyroid that was seen was thyroglossal cyst. The common presenting symptom was a painless midline neck swelling. In seven out of eight cases the cyst was located in the subhyoid region, while in one case it was in the suprahyoid region. In all these cases, the thyroid scan revealed no functioning thyroid tissue in the cyst and a normal thyroid gland.

Of the four cases of ectopic thyroid, three were lingual thyroids and one was seen in the subhyoid location, with absence of a normal thyroid gland. Two of the lingual thyroid cases presented with an incidentally noted swelling at the base of the tongue, while one presented with hypothyroid features.

**Figure 1:**
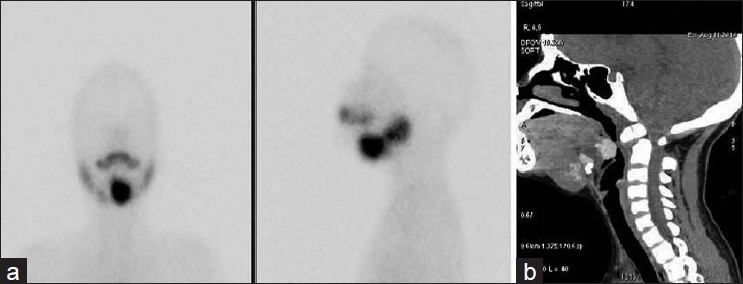
(a, b) Thyroid scan and CT image (sagittal section) showing dual ectopia – sublingual and suprahyoid – with absence of normal thyroid

**Figure 2:**
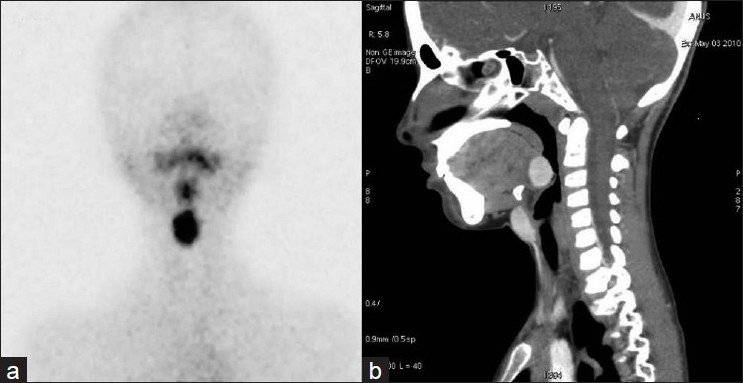
(a, b) Thyroid scan and CT image (sagittal section) showing dual ectopia - sublingual and subhyoid - with absence of normal thyroid

**Figure 3:**
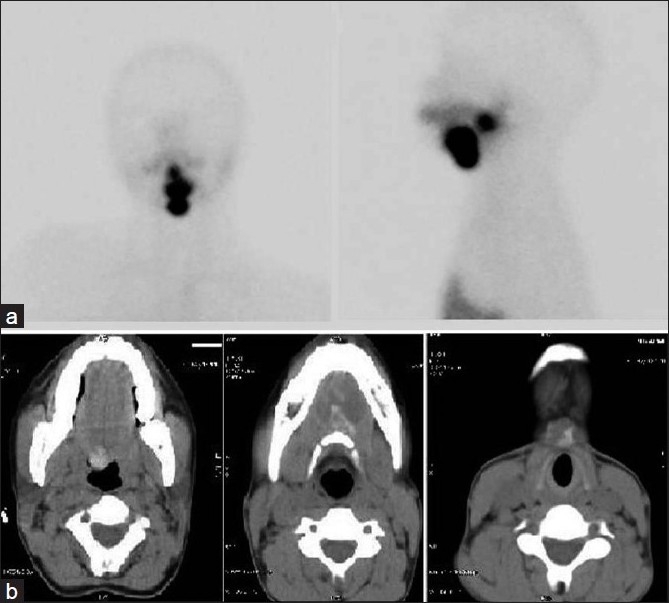
(a, b) Thyroid scan and CT image (axial section at different levels) showing triple ectopia - sublingual, suprahyoid, and subhyoid - with absence of normal thyroid

**Figure 4:**
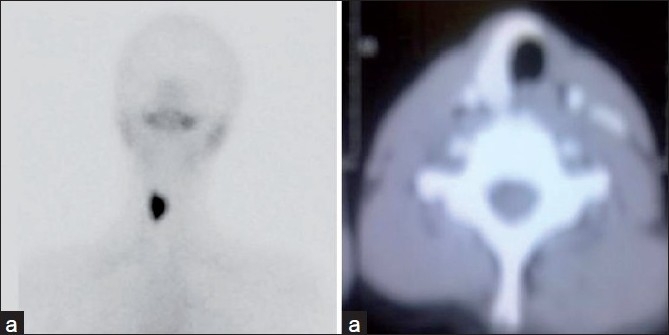
(a, b) Thyroid scan and CT image (axial section) showing thyroid hemiagenesis

In the four cases of dual ectopia in our series, ectopic thyroid tissue was found in the sublingual location in all the cases, with second ectopic tissue being found in the suprahyoid [Figure 1a and b] or subhyoid [Figure 2a and b] locations. The case of triple ectopia was a 20-year-old female who presented with a submandibular neck swelling. Her biochemical profile was suggestive of subclinical hypothyroidism. The thyroid scan revealed three areas of abnormal tracer uptake in the region of the base of the tongue and the suprahyoid and the subhyoid locations [Figure 3a]. CT images showed hyperdense soft tissue in the region of base of tongue and hyperdense tissue with cystic degeneration in suprahyoid and subhyoid locations [Figure 3b]. The patient with hemiagenesis presented with a neck swelling and a normal thyroid profile. The thyroid scan revealed normal tracer uptake in the right lobe of the thyroid and absent tracer uptake in the region of the left lobe of the thyroid [Figure 4a]. CT images confirmed the findings [Figure 4b].

## DISCUSSION

The main reason we present this retrospective analysis is to report the high incidence of rare developmental disorders of thyroid gland that we found in our practice. A review of literature (published in 2008) for dual ectpoic thyroid gland revealed that only 27 cases had been reported.[8] Since then one more case has been reported by McCoul *et al*.[16] There is only one case of triple ectopia (a 6-year-old boy with neck swelling, global developmental delay, and high serum TSH values) reported in the literature.[9] We report four cases of dual ectopia, which is 20% of the total developmental disorders of the thyroid gland we found in 5 months. We also report a case of triple ectopia in an otherwise healthy 20-year-old married female and mother of a healthy 2-year-old child. This patient, who presented with a long-standing submandibular swelling, is only the second case of triple ectopia to be reported. Her thyroid profile was suggestive of subclinical hypothyroidism. Our findings may indicate an increased incidence of multiple ectopia of thyroid gland due to some unknown reason or it may be that the condition has been under-reported previously.

More than 100 cases of thyroid hemiagenesis have been reported. The left lobe was absent in 80% of these patients.[3] The case of hemiagenesis of thyroid that we report presented with a swelling in the neck. The biochemical profile of this patient suggested euthyroid status. Thyroid scintigraphy and CT images revealed an absent left lobe of thyroid.

## CONCLUSION

This retrospective study emphasizes the indispensable role of thyroid scintigraphy in midline neck swellings of childhood. Almost all midline neck swellings of childhood should be evaluated with thyroid scintigraphy, along with other radiological and biochemical profiles, to plan the management.

Our experience may suggest an increase incidence of multiple ectopia of the thyroid gland. Further prospective studies are necessary to understand the etiopathogenesis and the exact incidence of the condition.
